# Supplementary material for: Meta-Analysis and Gene Set Enrichment Relative to ER Status Reveal Elevated Activity of MYC and E2F in the “Basal” Breast Cancer Subgroup
Source: PLoS One. 2009 Mar 9;4(3):e4710. doi: 10.1371/journal.pone.0004710 (PMC2650420; doi:10.1371/journal.pone.0004710)
Supplement: Dataset S1 — GSEA results from first screen. The file contains a zipped archive of the HTML results of the second GSEA screen. Reports for enriched or depleted datasets may be accessed from the “index.html” link within the my_analysis.GseaPreranked.1219975950625.rpt.zip ZIP archive. Enrichment plots are available for each of the 50 most enriched and depleted gene sets. (13.32 MB ZIP) [file pone.0004710.s001.zip › my_analysis.GseaPreranked.1219975950625.rpt.zip!//IDX_TSA_UP_CLUSTER3.html]

Details for gene set IDX\_TSA\_UP\_CLUSTER3[GSEA]

|  || Dataset | zSco.ERneg.to.ERpos\_collapsed |
| Phenotype | NoPhenotypeAvailable |
| Upregulated in class | na\_pos |
| GeneSet | IDX\_TSA\_UP\_CLUSTER3 |
| Enrichment Score (ES) | 0.687896 |
| Normalized Enrichment Score (NES) | 2.7671783 |
| Nominal p-value | 0.0 |
| FDR q-value | 0.0 |
| FWER p-Value | 0.0 |
Table: GSEA Results Summary

  

Fig 1: Enrichment plot: IDX\_TSA\_UP\_CLUSTER3      
 Profile of the Running ES Score & Positions of GeneSet Members on the Rank Ordered List

  

| PROBE | GENE SYMBOL | GENE\_TITLE | RANK IN GENE LIST | RANK METRIC SCORE | RUNNING ES | CORE ENRICHMENT || 1 | CDC20 |  |  | 9 | 7.151 | 0.0241 | Yes |
| 2 | TTK |  |  | 11 | 7.059 | 0.0485 | Yes |
| 3 | LMO4 |  |  | 23 | 6.717 | 0.0710 | Yes |
| 4 | MCM5 |  |  | 31 | 6.533 | 0.0931 | Yes |
| 5 | BUB1 |  |  | 41 | 6.445 | 0.1148 | Yes |
| 6 | NASP |  |  | 59 | 6.201 | 0.1350 | Yes |
| 7 | KLF5 |  |  | 87 | 5.966 | 0.1537 | Yes |
| 8 | TRIP13 |  |  | 96 | 5.890 | 0.1735 | Yes |
| 9 | MKI67 |  |  | 98 | 5.871 | 0.1938 | Yes |
| 10 | CDC45L |  |  | 108 | 5.824 | 0.2133 | Yes |
| 11 | KIF2C |  |  | 124 | 5.703 | 0.2319 | Yes |
| 12 | CX3CL1 |  |  | 169 | 5.477 | 0.2476 | Yes |
| 13 | MCM4 |  |  | 180 | 5.436 | 0.2657 | Yes |
| 14 | CDCA8 |  |  | 194 | 5.328 | 0.2832 | Yes |
| 15 | MCM10 |  |  | 201 | 5.315 | 0.3012 | Yes |
| 16 | USP1 |  |  | 222 | 5.212 | 0.3178 | Yes |
| 17 | BIRC5 |  |  | 229 | 5.191 | 0.3353 | Yes |
| 18 | CKS2 |  |  | 239 | 5.145 | 0.3525 | Yes |
| 19 | TUBB6 |  |  | 264 | 5.044 | 0.3682 | Yes |
| 20 | KIF4A |  |  | 266 | 5.031 | 0.3855 | Yes |
| 21 | RAD51AP1 |  |  | 267 | 5.028 | 0.4030 | Yes |
| 22 | MCM7 |  |  | 289 | 4.970 | 0.4186 | Yes |
| 23 | EZH2 |  |  | 312 | 4.888 | 0.4339 | Yes |
| 24 | KIF20A |  |  | 318 | 4.874 | 0.4504 | Yes |
| 25 | NCAPH |  |  | 348 | 4.799 | 0.4649 | Yes |
| 26 | CKS1B |  |  | 416 | 4.638 | 0.4759 | Yes |
| 27 | CCNB2 |  |  | 431 | 4.589 | 0.4908 | Yes |
| 28 | AURKA |  |  | 460 | 4.483 | 0.5042 | Yes |
| 29 | MAD2L1 |  |  | 477 | 4.446 | 0.5184 | Yes |
| 30 | RAD51 |  |  | 508 | 4.332 | 0.5312 | Yes |
| 31 | DLG7 |  |  | 521 | 4.306 | 0.5452 | Yes |
| 32 | PRC1 |  |  | 543 | 4.253 | 0.5584 | Yes |
| 33 | MCM2 |  |  | 630 | 4.080 | 0.5660 | Yes |
| 34 | SMC2 |  |  | 651 | 4.028 | 0.5785 | Yes |
| 35 | MCM3 |  |  | 656 | 4.019 | 0.5921 | Yes |
| 36 | TACC3 |  |  | 669 | 3.995 | 0.6051 | Yes |
| 37 | CSRP2 |  |  | 706 | 3.916 | 0.6159 | Yes |
| 38 | KIF11 |  |  | 713 | 3.909 | 0.6290 | Yes |
| 39 | TFDP1 |  |  | 750 | 3.843 | 0.6397 | Yes |
| 40 | FEN1 |  |  | 754 | 3.838 | 0.6527 | Yes |
| 41 | TYMS |  |  | 829 | 3.738 | 0.6601 | Yes |
| 42 | NUSAP1 |  |  | 836 | 3.732 | 0.6726 | Yes |
| 43 | PLK1 |  |  | 988 | 3.499 | 0.6733 | Yes |
| 44 | E2F8 |  |  | 1122 | 3.334 | 0.6749 | Yes |
| 45 | CDC2 |  |  | 1259 | 3.182 | 0.6756 | Yes |
| 46 | ASF1B |  |  | 1319 | 3.119 | 0.6820 | Yes |
| 47 | HELLS |  |  | 1382 | 3.057 | 0.6879 | Yes |
| 48 | CCNB1 |  |  | 1626 | 2.823 | 0.6793 | No |
| 49 | ILF2 |  |  | 1644 | 2.807 | 0.6878 | No |
| 50 | RRM2 |  |  | 2047 | 2.485 | 0.6660 | No |
| 51 | ADAM8 |  |  | 2347 | 2.288 | 0.6514 | No |
| 52 | SLBP |  |  | 2921 | 1.974 | 0.6149 | No |
| 53 | CDC6 |  |  | 3185 | 1.847 | 0.6015 | No |
| 54 | CCNF |  |  | 3265 | 1.809 | 0.6018 | No |
| 55 | CCNE2 |  |  | 3424 | 1.738 | 0.5959 | No |
| 56 | TOPBP1 |  |  | 3475 | 1.722 | 0.5981 | No |
| 57 | SLCO3A1 |  |  | 3563 | 1.679 | 0.5973 | No |
| 58 | PTGFR |  |  | 3846 | 1.573 | 0.5815 | No |
| 59 | HMGB2 |  |  | 3883 | 1.558 | 0.5842 | No |
| 60 | POLA1 |  |  | 3967 | 1.525 | 0.5832 | No |
| 61 | CSTF2 |  |  | 3979 | 1.518 | 0.5876 | No |
| 62 | RFC5 |  |  | 4034 | 1.500 | 0.5887 | No |
| 63 | RACGAP1 |  |  | 4097 | 1.475 | 0.5892 | No |
| 64 | ABCA1 |  |  | 4273 | 1.403 | 0.5808 | No |
| 65 | DTL |  |  | 4509 | 1.314 | 0.5676 | No |
| 66 | KIAA0101 |  |  | 4770 | 1.230 | 0.5523 | No |
| 67 | RRM1 |  |  | 4872 | 1.197 | 0.5488 | No |
| 68 | HGF |  |  | 4931 | 1.177 | 0.5485 | No |
| 69 | INCENP |  |  | 5907 | 0.845 | 0.4778 | No |
| 70 | LRP5 |  |  | 6202 | 0.750 | 0.4582 | No |
| 71 | PRIM1 |  |  | 6525 | 0.642 | 0.4361 | No |
| 72 | OSMR |  |  | 7162 | 0.430 | 0.3895 | No |
| 73 | AQP1 |  |  | 7776 | 0.199 | 0.3439 | No |
| 74 | KIF22 |  |  | 8725 | -0.180 | 0.2729 | No |
| 75 | BRCA1 |  |  | 8777 | -0.199 | 0.2697 | No |
| 76 | DCK |  |  | 8935 | -0.280 | 0.2589 | No |
| 77 | NUP85 |  |  | 10482 | -1.147 | 0.1460 | No |
| 78 | WNT4 |  |  | 11278 | -1.809 | 0.0923 | No |
| 79 | IL13RA1 |  |  | 11641 | -2.160 | 0.0724 | No |
| 80 | GAS6 |  |  | 12028 | -2.598 | 0.0523 | No |
| 81 | NQO1 |  |  | 12453 | -3.182 | 0.0313 | No |
| 82 | IMPDH2 |  |  | 13108 | -4.860 | -0.0013 | No |
| 83 | MAN2A1 |  |  | 13136 | -4.965 | 0.0139 | No |
Table: GSEA details [plain text format]

  

Fig 2: IDX\_TSA\_UP\_CLUSTER3: Random ES distribution      
 Gene set null distribution of ES for **IDX\_TSA\_UP\_CLUSTER3**
